# Anaerobic microbial community response to methanogenic inhibitors 2‐bromoethanesulfonate and propynoic acid

**DOI:** 10.1002/mbo3.349

**Published:** 2016-03-14

**Authors:** Tara M. Webster, Adam L. Smith, Raghav R. Reddy, Ameet J. Pinto, Kim F. Hayes, Lutgarde Raskin

**Affiliations:** ^1^ Civil & Environmental Engineering DepartmentUniversity of MichiganAnn ArborMichigan; ^2^ Infrastructure and Environment Research DivisionSchool of EngineeringUniversity of GlasgowGlasgowUnited Kingdom; ^3^ Present address: TMW Soil and Crop Sciences SectionCornell UniversityIthacaNew York; ^4^ Present address: ALS Astani Department of Civil and Environmental EngineeringUniversity of Southern CaliforniaLos AngelesCalifornia; ^5^ Present address: AJP Department of Civil and Environmental EngineeringNortheastern UniversityBostonMassachusetts

**Keywords:** 16S rRNA, 2‐bromoethanesulfonate, *mcrA*, methanogenic inhibitors, propynoic acid.

## Abstract

Methanogenic inhibitors are often used to study methanogenesis in complex microbial communities or inhibit methanogens in the gastrointestinal tract of livestock. However, the resulting structural and functional changes in archaeal and bacterial communities are poorly understood. We characterized microbial community structure and activity in mesocosms seeded with cow dung and municipal wastewater treatment plant anaerobic digester sludge after exposure to two methanogenic inhibitors, 2‐bromoethanesulfonate (BES) and propynoic acid (PA). Methane production was reduced by 89% (0.5 mmol/L BES), 100% (10 mmol/LBES), 24% (0.1 mmol/LPA), and 95% (10 mmol/LPA). Using modified primers targeting the methyl‐coenzyme M reductase (*mcrA*) gene, changes in *mcrA* gene expression were found to correspond with changes in methane production and the relative activity of methanogens. Methanogenic activity was determined by the relative abundance of methanogen 16S rRNA cDNA as a percentage of the total community 16S rRNA cDNA. Overall, methanogenic activity was lower when mesocosms were exposed to higher concentrations of both inhibitors, and aceticlastic methanogens were inhibited to a greater extent than hydrogenotrophic methanogens. Syntrophic bacterial activity, measured by 16S rRNA cDNA, was also reduced following exposure to both inhibitors, but the overall structure of the active bacterial community was not significantly affected.

## Introduction

Methane can be viewed as a potent greenhouse gas, an energy source, a dangerous, and explosive byproduct of anaerobic biodegradation, a waste product diverting energy from animal feed, or a driver of microbial carbon cycling (Hallam et al. 2003; Dupont and Accorsi 2006; Knittel and Boetius 2009; Appels et al. 2011; Chowdhury and Dick 2013; IPCC 2013; Patra and Yu 2013). Due to the importance of methane in fields ranging from climate science to animal husbandry, much research has focused on understanding the activity of methanogenic archaea under anaerobic conditions (Reeve et al. 1997; Conrad 2007). Aerobic methane generation has also been identified and may be an important source of methane from oceans (Karl et al. 2008); however, this study focuses on methane production under anaerobic conditions. All known methanogenic archaea contain genes that encode for the methyl‐coenzyme M reductase (MCR), which catalyzes the final step of methanogenesis. There are two isoenzymes, MCRI and MCRII, and the *mcrA* and *mrtA* genes encode for the *α*‐subunit of each of these isoenzymes, respectively (Reeve et al. 1997). The *mcrA*/*mrtA* genes have been a common target for measuring methanogen abundance, activity, and diversity. Distinctions between *mcrA* and *mrtA* genes often are not made in the literature and hereafter we use *mcrA* to refer to the combination of both genes, unless specified otherwise. The agreement between phylogenetic trees based on 16S rRNA genes and *mcrA* genes has helped to support the use of the *mcrA* gene as a methanogen‐specific phylogenetic target (Luton et al. 2002).

Compounds that inhibit methanogenesis have been important in research to study pure cultures of methanogens (Ungerfeld et al. 2004; Watkins et al. 2012), carbon cycling in soils (Sugimoto and Wada 1993; Wu et al. 2001), ruminal methanogens (Ungerfeld et al. 2006; Zhou et al. 2011b), dechlorination (Perkins et al. 1994; Chiu and Lee 2001), mercury methylation (Han et al. 2010; Avramescu et al. 2011), production of volatile fatty acids (Zhang et al. 2013; Jung et al. 2015), anaerobic digestion (Zinder et al. 1984; Navarro et al. 2014), and the degradation of nitrosamines (Tezel et al. 2011) and methanethiol (Sun et al. 2015). Further, inhibitors have been useful in elucidating the activity of methanogens related to metal and metalloid methylation (Meyer et al. 2008; Thomas et al. 2011). A variety of chemicals have been applied to inhibit methanogenesis in livestock to either reduce methane emissions or to direct more of the feed energy to animals for increased agricultural output (i.e., milk and meat) (Machmüller and Kreuzer 1999; Boadi et al. 2004; Beauchemin et al. 2009). Regardless of the intended use, when methanogenic inhibitors are used in mixed communities, detailed characterization of inhibitor‐induced changes to both archaeal and bacterial populations is needed to ensure that the observed effects can be accurately ascribed to the inhibition of methanogenic activity and to elucidate any indirect effects. This is especially important given that a wide diversity of methanogenic inhibitors with varying properties and mechanisms of action are available. Methanogenic inhibitors can be divided into several categories (as reviewed by (Liu et al. 2011)), including analogs of coenzyme M (Gunsalus et al. 1978; Zinder et al. 1984), inhibitors of methanopterin biosynthesis (Dumitru et al. 2003), medium‐ and long‐chain fatty acids (Prins et al. 1972; Soliva et al. 2003), nitrocompounds (Zhou et al. 2011b), halogenated hydrocarbons (Denman et al. 2007), ethylene (Oremland and Taylor 1975), acetylene (Oremland and Taylor 1975; Sprott et al. 1982), and unsaturated analogs of propionate and butyrate (Ungerfeld et al. 2003, 2004, 2006; Zhou et al. 2011b).

While many inhibitors are considered methanogen‐specific, various studies have found that other microorganisms can be affected. The most commonly used methanogenesis inhibitor, 2‐bromoethanesulfonate (BES), a coenzyme M analog, has been found to also inhibit dechlorinating bacteria (Loffler et al. 1997; Chiu and Lee 2001) and to affect bacterial growth on aliphatic alkenes (Boyd et al. 2006). Propynoic acid (PA), an unsaturated propionate analog with one triple carbon bond, is also an effective inhibitor of methanogenesis (Ungerfeld et al. 2004; Zhou et al. 2011b). However, limited studies have been performed on the effects of PA on the structure of microbial communities (Patra and Yu 2013). To date, studies of the impacts of methanogenic inhibitors on bacterial and archaeal communities have relied on clone libraries, denaturing gradient gel electrophoresis (DGGE), or terminal restriction fragment length polymorphism (TRFLP) targeting the 16S rRNA gene (Chiu and Lee 2001; Xu et al. 2010a,b; Patra and Yu 2013; Lins et al. 2015) and the *mcrA* gene (Denman et al. 2007). Results from DGGE‐based evaluations of the impact of inhibitors have shown changes in the overall community structure, but did not yield insights into how specific populations were impacted (Chiu and Lee 2001; Patra and Yu 2013). Studies using TRFLP and clone libraries of the 16S rRNA gene have reported decreases in the relative abundance of aceticlastic methanogens and syntrophic bacteria and increases in the relative abundance of homoacetogens after exposure of mesophilic anaerobic digester sludge to BES and chloroform (Xu et al. 2010a,b). In a study of cow rumen communities, *mcrA* gene clone libraries and quantitative PCR revealed a decrease in the most abundant methanogenic genus, *Methanobrevibacter*, under BES inhibited conditions (Denman et al. 2007). Since these studies relied on DNA‐based techniques (Chiu and Lee 2001; Denman et al. 2007; Xu et al. 2010a,b; Patra and Yu 2013; Lins et al. 2015), they may not have revealed short‐term changes in microbial activity in batch mesocosms or in systems with low yield, because of low growth rates and the retention of dead or inactive biomass and extracellular DNA (Chiao et al. 2014; Smith et al. 2015a).

In this study, we evaluated a modification to commonly used PCR primer sets targeting the *mcrA* gene to expand their coverage. We then applied this primer set to track the expression of *mcrA* genes by using reverse transcriptase quantitative PCR (RT‐qPCR) in mixed communities seeded with anaerobic digester sludge and cow dung at different levels of inhibition by either BES or PA. The effects of BES and PA on methanogenic and bacterial populations were characterized through a combination of DNA‐ and RNA‐based Illumina sequencing targeting the V4 region of the 16S rRNA gene and 16S rRNA cDNA, and the *mcrA* gene and *mcrA* transcript cDNA.

## Experimental Procedures

### Primer design and mock community construction

Primers targeting the *mcrA* gene were designed through an in silico analysis followed by testing with pure cultures and mock communities. First, existing primer sets (Juottonen et al. 2006; Steinberg and Regan 2008, 2009; Zeleke et al. 2013) were compared to partial *mcrA* sequences downloaded from GenBank (NCBI, Bethesda, MD) and back‐translated full‐length McrA protein sequences using EMBOSS Backtranseq with the *Methanothermobacter thermoautotrophicus* strain Delta H codon usage Table (EMBL EBI, Hinxton, UK) using MEGA 6.0 (Tamura et al. 2013). The forward primer mlas (Steinberg and Regan 2008) was modified with additional degeneracies (5′GGYGGTGTMGG**N**TTCACHCARTA‐3′ bold font indicates changes). The reverse primer mcrA‐rev was used as reported previously (5′‐CGTTCATBGCGTAGTTVGGRTAGT‐3′) (Steinberg and Regan 2008). Primer specificity and coverage were assessed in silico using MFE primer 2.0 (Qu et al. 2012). The V4 region of 16S rRNA gene was targeted using universal primers F515 (5′‐ GTGCCAGCMGCCGCGGTAA‐3′) and R806 (5′‐ GGACTACHVGGGTWTCTAAT‐3′) (Caporaso et al. 2011). The coverage of these primers was verified with TestPrime 1.0 (Klindworth et al. 2012). Both primer sets were checked for complementarity with sequences from the complete genomes of the methanogens used in the mock communities (Table S1).

To verify the amplification of the *mcrA* gene from a range of methanogens using the redesigned primers, DNA extracts from pure cultures of methanogens were used as a template for PCR over a range of annealing temperatures. PCR was performed using 20 *μ*L reactions with primers at 500 nmol/L, 0.5 ng of template, 0.3 mg/mL bovine serum albumin (BSA), 10 *μ*L Phusion High Fidelity Master Mix (NEB, Ipswich, MA), and nuclease‐free water. An initial 2 min denaturation at 95°C was followed by 30 cycles of denaturing at 95°C for 20 sec, annealing at 55°C for 15 sec, and extension at 72°C for 30 sec, with a final extension at 72°C for 5 min. PCR products were visualized on a 1.5% agarose gel.

**Figure 1:**
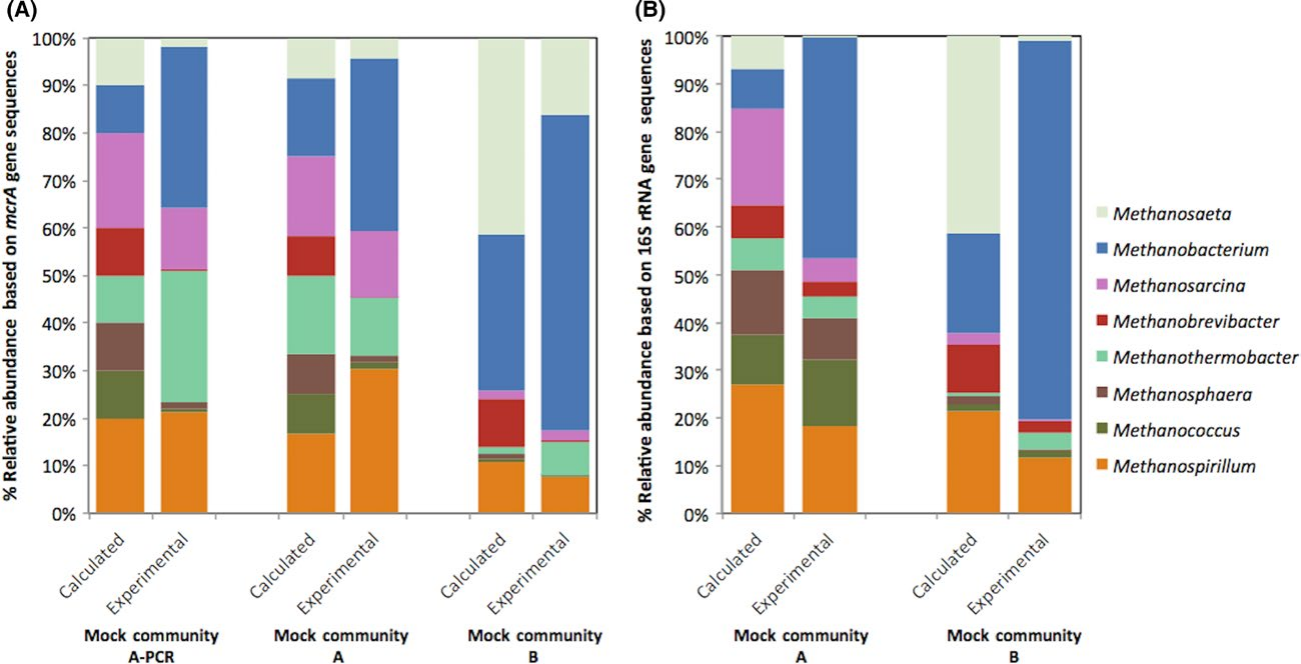
Comparison between the calculated and experimental relative abundance of methanogen mock communities based on the sequencing of the *mcrA*gene (A) and 16S rRNA gene (B). Mock community A‐PCR was created by pooling the PCR products from individually amplified *mcrA *genes for each methanogen. Mock community A and B were created by pooling DNA extracts before amplification. The expected compositions were calculated based on DNA concentrations of the extracts from 10 strains measured by fluorospectrometry, genome size, and gene copy number, or PCR product quantification by fluorospectrometry (Table S2). Two different strains were included for the genera *Methanospirillum *and Methanosarcina. All strains included in the mock communities were identified through *mcrA *gene and 16S rRNA gene sequencing. The *mcrA *gene‐based sequencing results included one sequence each identified as *Methanohalophilus *and *Methanoculleus*, which were excluded from the graphs.

Three different mock communities were created by mixing varying amounts of either DNA extracts or amplified PCR products. Mock community A was made by mixing DNA extracted from 10 methanogenic strains based on concentration and genome length to achieve a relatively even community; the inclusion of two *Methanospirillum* and *Methanosarcina* strains and differential gene copy numbers contribute to slight deviations from complete evenness. Mock community A‐PCR was made by mixing *mcrA* gene amplified PCR products from each methanogen based on PCR product concentration to achieve a community similar to mock community A. Mock community B was constructed by mixing DNA extracts from each methanogen based on genome length to achieve a community representative of an anaerobic digester (Smith et al. 2013). Expected community structures based on these calculations are shown in Figure 1. Samples from these mock communities were submitted for sequencing and analyzed as described below.

### Mesocosm set‐up and sampling

Mesocosms were seeded by mixing 6 g wet cow dung, collected from a field where grass and corn fed cattle were grazing using sterile plastic scoops, with 100 mL of concentrated (approximately 5000 mg/L total suspended solids) anaerobic digester sludge collected from a mesophilic (32°C) wastewater treatment plant anaerobic sludge digester (Northfield Wastewater Treatment Plant, Whitmore Lake, MI) in 150‐mL serum bottles. Control mesocosms contained no added inhibitor. The effect of 2‐bromoethanesulfonate (BES) addition was evaluated at concentrations of 0.5 and 10 mmol/L, whereas propynoic acid (PA) was tested at concentrations of 0.1 and 10 mmol/L. Duplicate mesocosms were run for the control without inhibitor, 10 mmol/L BES, and 10 mmol/L PA conditions, and single mesocosms were run for 0.5 mmol/L BES, 50 mmol/L BES, 0.1 mmol/L PA, and 2 mmol/L PA. Excellent agreement in gas production was observed in duplicate mesocosms (Fig. S1). The starting pH ranged from 6.3 to 7.0 in the mesocosms and was adjusted to pH 7.0 using sodium hydroxide prior to capping with a butyl rubber stopper, crimp sealing, and purging with N_2_ gas. Incubations were carried out in a 31°C water bath and the mesocosm contents were mixed on magnetic stir plates.

A glass syringe (Chemglass Life Sciences, Vineland, New Jersey) was used to measure gas production and collect gas for composition measurements about every other day. The CH_4_, CO_2_, and N_2_ composition in the headspace gas was measured in duplicate for each sample using a gas chromatograph (Gow‐Mac, Bethlehem, PA) coupled with a thermal conductivity detector (TCD). On day 9, after a final collection of the headspace gas, the bottles were opened and the biomass was centrifuged at 4°C. The supernatant was decanted and biomass samples were collected for DNA and RNA extractions, the latter being preserved with RNAlater (Qiagen, Valencia, CA). Following collection, biomass samples were frozen at −80°C until extraction.

### Mesocosm nucleic acid extractions, cDNA synthesis, and quantitative PCR

Duplicate DNA and RNA extractions were performed for duplicate biomass samples collected from the same mesocosm for the following conditions: control, 0.5 mmol/L BES, 10 mmol/L BES, 0.1 mmol/L PA, and 10 mmol/L PA. The automated extraction Maxwell 16 Blood LEV (Promega, Madison, WI) kit or Maxwell 16 simplyRNA tissue kit, for DNA or RNA, respectively, was used according to the manufacturer's instructions with slight modifications as described below. Briefly, zirconium beads (0.1 mm) and lysis buffer were added to each sample and three 2‐min bead beating steps were performed, replacing the lysis buffer after each bead beating. Proteinase K was added to each sample for DNA extraction prior to the automatic extraction steps. For RNA extraction, the method was the same, except bead beating was performed in 1‐thioglycerol homogenization buffer and 10 *μ*L of DNase 1 was added to the extraction kit. Nucleic acid quality and quantity were determined using spectrophotometry (Nanodrop 1000, Thermo Fischer Scientific, Wilmington, DE), fluorospectrometry (Quantifluor dsDNA and RNA systems (Promega, Madison, WI)), and for RNA samples using electrophoresis with the Experion RNA analysis kit (Bio‐Rad, Hercules, CA). cDNA was synthesized using SuperScript^®^ VILO cDNA synthesis kit according to the manufacturer's instructions (Invitrogen, Carlsbad, CA).

PCR products for use as qPCR standards were generated using the protocol described above for both *mcrA* and 16S rRNA gene amplicons, using DNA extracts from mesocosm samples pooled by equal mass as the template (He and Mcmahon 2011; Sonthiphand et al. 2013). PCR products were visualized on a 1.5% agarose gel and the band was excised and purified with the QIAquick Gel Extraction Kit (Qiagen). Amplified and purified pools were quantified using the Quantifluor dsDNA system and fluorospectrometry. Serial dilutions of the pools were prepared for qPCR standards and ranged from 10^7^–10^2^ copies/*μ*L for *mcrA* and 10^8^–10^4^ copies/*μ*L for 16S rRNA genes. The Mastercycler Realplex Ep (Eppendorf, Hamburg, Germany) was used to perform RT‐qPCR with triplicate wells for each sample and reaction volumes of 19 *μ*L using Fast Plus EvaGreen Master Mix (Biotium, Hayward, CA). Forward and reverse primer concentrations were 500 nmol/L, except the reverse *mcrA* primer was used at 250 nmol/L. The conditions used for thermocycling were as described above with slight modifications. Instead of 30 cycles, 50 cycles were used and a melting curve analysis was performed as the final step to check for spurious amplification products. To improve annealing conditions for the *mcrA* transcript cDNA quantification, an initial 2 min denaturation of the cDNA at 95°C was followed by five cycles of 95°C for 20 sec, 55°C for 15 sec, followed by a temperature ramp of 0.1°C per sec to 72°C (Luton et al. 2002; Morris et al. 2014), and extension for 72°C for 30 sec. Then, 45 cycles were performed without the temperature ramp with a final extension at 72°C for 5 min. The standard curves *R* ^*2*^ were 0.995 and 0.998 and efficiencies were 74% and 89%, for *mcrA* and 16S rRNA genes, respectively.

### Sequencing and analysis

Samples from the mock community, mesocosm DNA, and mesocosm cDNA were submitted for sequencing of the V4 region of the 16S rRNA gene at the Host Microbiome Initiative (University of Michigan, Ann Arbor, MI). Primers F515 and R806 (Caporaso et al. 2011) were modified for dual‐index sequencing as described by Kozich et al. (2013). PCR was performed using Accuprime TAQ (Invitrogen) and thermocycling conditions were 95**°**C denaturation for 2 min, followed by 30 cycles of denaturation at 95**°**C for 20** **sec, annealing at 55**°**C for 15** **sec, and extension at 72**°**C for 5 min, the final extension was performed at 72**°**C for 5 min. Samples were also submitted for sequencing of the *mcrA* gene following the amplification procedure described above. After amplification of either gene, the SequalPrep Normalization Plate Kit (Life Technologies, Grand Island, NY) was used to pool samples by equal mass. Amplicons were multiplexed and sequenced using the Illumina MiSeq, Reagent Kit V2 was used for *mcrA* amplicons resulting in a total of 20,842 paired‐end reads after quality filtering, and between 193 and 2240 sequences per sample. For 16S rRNA gene amplicons, Reagent Kit V3 was used and resulted in 15,152 sequences per sample after quality filtering and subsampling. The resulting sequences were processed with mothur (Schloss et al. 2009) following the Schloss MiSeq SOP (Kozich et al. 2013) and classified using the 16S rRNA taxonomy from the Ribosomal Database Project (Cole et al. 2013) and the *mcrA* taxonomic database from Yang et al. (2014). For *mcrA* sequences, four ambiguous base pairs were allowed and a similarity cutoff of 85.8% was used for the genus level corresponding to a 97% cutoff for the 16S rRNA (Yang et al. 2014). The generated sequence data were submitted to the DDBJ/EMBL/GenBank databases under Accession Number SRP062486.

## Results and Discussion

### *mcrA* primer design and mock community characterization

To target the *mcrA* gene in methanogens, the mlas forward primer described by Steinberg and Regan (2009) was modified with additional degeneracies and used with the previously reported mcrA‐rev reverse primer (Steinberg and Regan 2008). These modifications improved the predicted amplification for 10 of the 32 methanogens with complete genomes available (Table S1). Amplification was confirmed using 10 DNA extracts from pure cultures of methanogens (Tables S2, S3). These DNA extracts were pooled to create two mock communities A and B, to represent either a relatively even community (A) or an uneven community (B) with relative methanogen DNA abundances similar to those found in an anaerobic digester (Smith et al. 2013). For mock communities A and B, both the 16S rRNA genes and *mcrA* genes were sequenced. A third mock community, mock community A‐PCR was created by pooling the PCR products from individually amplified *mcrA* genes for each methanogen. Calculated relative abundances were determined based on pooled concentrations and the experimental sequencing results are compared in Figure 1.

When comparing the results obtained for mock communities A and B, the trends were similar for both genes although some differences in the percent relative abundances were observed (Fig. 1). A previous comparison of methanogen mock communities with TRFLP noted greater differences between expected and observed communities based on the *mcrA* gene as compared to the 16S rRNA gene, which were attributed to the higher number of degeneracies in the primers used for the *mcrA* gene (Lueders and Friedrich 2003). Comparing our calculated and experimentally measured communities using the *θ*_yc_ community dissimilarity metric, we observed a lower community dissimilarity based on the *mcrA* gene (*θ*_yc_ of 0.48, 0.33, and 0.40 for mock communities A‐PCR, A, and B, respectively) compared to the dissimilarity based on the 16S rRNA gene (*θ*_yc_ of 0.58 and 0.72 for mock communities A and B, respectively). These differences may result, in part, from challenges in quantification using amplicon sequencing due to gene target‐specific biases, PCR conditions, quantification method, and primers used (Suzuki and Giovannoni 1996; Zhou et al. 2011a; Pinto and Raskin 2012).

The relative abundance of *Methanobacterium* was much greater, while the relative abundance of *Methanosaeta* was much lower than predicted for both the 16S rRNA and *mcrA* genes (Fig. 1). However, both genera were more abundant in mock community B compared to mock community A for both genes, as expected. For *Methanobrevibacter, Methanococcus,* and *Methanosphaera*, the relative abundance as measured by the *mcrA* gene was much lower in mock communities A and B as compared to the predicted values and those measured by the 16S rRNA gene. Obvious PCR biases were not responsible for this underrepresentation as the primers have no mismatches with their target sequences for these organisms (Table S3) and mock community A‐PCR, which was generated by pooling individually amplified PCR products of the *mcrA* gene from each strain, exhibited similar results (Fig. 1). Other factors that can affect sequencing errors include template concentration (Kennedy et al. 2014) and library preparation method (Schirmer et al. 2015). Errors during Illumina sequencing can be related to certain motifs, which can vary based on library preparation method (Schirmer et al. 2015). The differences between the predicted and the experimental sequencing results observed for the mock communities can be useful in guiding the analyses of mesocosm samples, as described below. Previous studies that compared the methanogen community structures using sequencing of the 16S rRNA gene, *mcrA* gene, and other functional genes related to methanogenesis have found some quantitative differences depending on the gene sequenced (Dziewit et al. 2015; Wilkins et al. 2015), but did not include mock communities for comparison. Given the observations made with the mock communities, we note that our interpretation of sequencing results from unknown mesocosm samples focuses on the comparison of relative abundances between samples.

### Inhibition reduced methane production, *mcrA* expression, and 16S rRNA of methanogens

**Figure 2:**
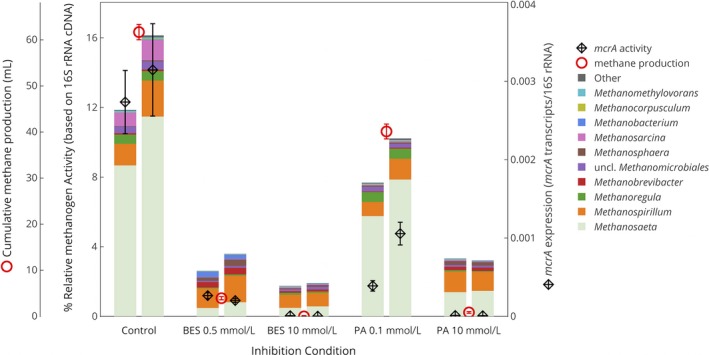
Cumulative methane production and molecular characterization of methanogens in cow dung and anaerobic digester sludge mesocosms after 9 days of incubation. Relative methanogen activity based on methanogen 16S rRNA cDNA as a % of the total community (including *Bacteria* and *Archaea*) (bars), *mcrA* expression normalized by 16S rRNA cDNA (diamonds) determined with RT‐qPCR, and cumulative methane production (circles). Error bars for methane production volume represent the propagated uncertainty in methane concentration measurements. *mcrA* expression is displayed as the averages and standard deviations of triplicate RT‐qPCR reactions. Duplicates shown represent duplicate biomass samples from the same reactors. No inhibitor was added in control conditions.

To characterize short‐term changes in mixed communities induced by methanogenic inhibitors, biomass samples were collected from cow dung and anaerobic digester sludge mesocosms operated for 9 days at varying levels of methanogenic activity controlled through the addition of BES and PA. Methanogenic activity was monitored through the measurement of methane production and *mcrA* gene expression. The microbial communities and their activities were characterized using sequencing of the 16S rRNA gene, 16S rRNA cDNA, *mcrA* genes, and *mcrA* transcript cDNA. As expected, with increasing concentrations of the methanogen inhibitors BES and PA, the rate of methane production and cumulative methane produced decreased (Figs. 2 and S1). Expression of the *mcrA* gene corresponded to the rate of methane production (Fig 2). This finding is important, as relationships between the expression of genes and the resulting function are often assumed but rarely confirmed (Rocca et al. 2015). Similarly, higher total methane production was associated with a higher proportion of active methanogens as measured by 16S rRNA cDNA sequences (referred to here as “relative activity”) of methanogens over the total community (including *Bacteria* and *Archaea*) (Fig. 2). This finding is consistent with other observations linking these measurements in an anaerobic membrane bioreactor (Smith et al. 2015b) and anaerobic digesters (Wilkins et al. 2015). There are well‐recognized biases associated with quantifying 16S rRNA cDNA to measure activity, including differences in *rrn* operon copy numbers and lifestyle strategies among different populations. These biases highlight the importance of comparing rRNA levels with other measures of metabolic activity (Blazewicz et al. 2013). Here, the observed correlation between methanogen 16S rRNA cDNA concentrations and expression levels of a functional gene specific to methanogens (Pearson matrix correlation *r *=* *0.93) (Fig. 2) indicates that 16S rRNA activity can be a reliable metric for methanogen activity, at least for the current conditions.

**Figure 3:**
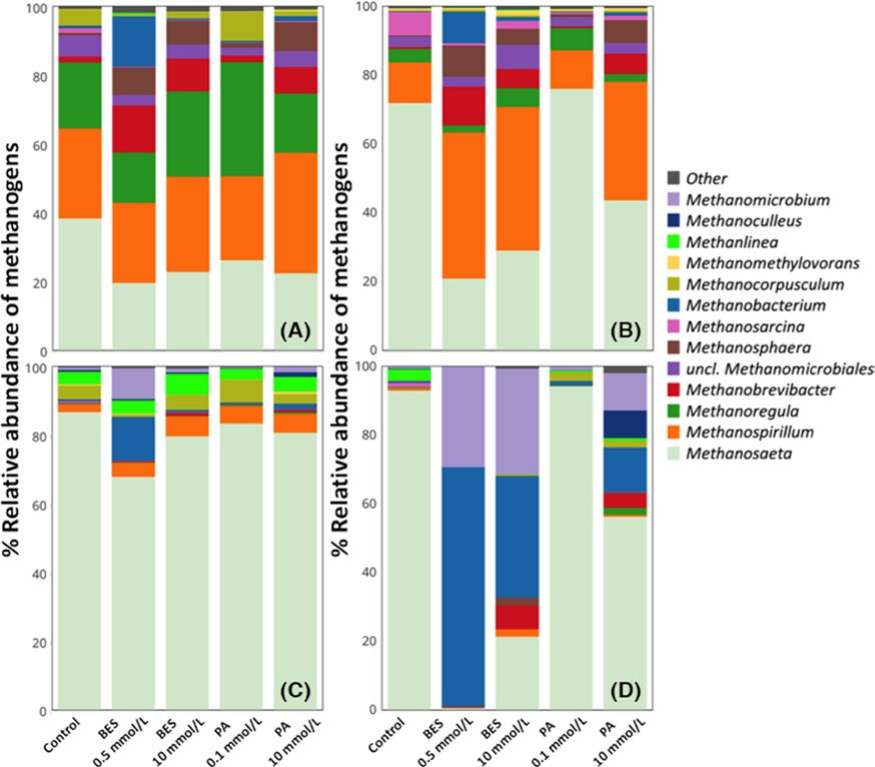
Relative abundance (DNA) and activity (RNA) of methanogens in anaerobic mesocosms after 9 days of incubation based on 16S rRNA genes (A), 16S rRNA cDNA (B), *mcrA* genes (C), and *mcrA* transcript cDNA (D), sequencing. Sequences from duplicate samples for each condition are combined (duplicates are shown in Figure S4).

Differences in the mesocosms for different inhibition conditions were evaluated by sequencing the 16S rRNA gene, 16S rRNA cDNA, *mcrA* gene, and *mcrA* transcript cDNA. As expected, given the short duration of the experiment, differences in the archaeal DNA‐based sequencing results for the five conditions were modest (Fig. 3A and C). In contrast, the RNA‐based sequencing results (Figure 3B and D), revealed substantial differences in the five mesocosms. These results highlight changes to the methanogenic community structure, but do not reflect changes in absolute abundance or activity. Based on the 16S rRNA cDNA quantification (Fig. 2), the methanogenic community was shown to become less active with increasing inhibitor concentration. As with the mock communities, the broad trends in relative abundance and activity across inhibition conditions within a given methanogenic genus were similar for the two different genes sequenced (Fig. 3A and B compared to Fig. 3C and D). However, the actual values for percent relative abundance and activity for the two genes were quite different. Similar to the results from the mock communities, *Methanosaeta* spp. appeared to be more abundant and active when *mcrA*‐based sequencing was used, while *Methanospirillum* spp. were more abundant and active according to 16S rRNA‐based sequencing.

*Methanosaeta* spp. were the most abundant and active methanogens in the control samples, representing 38% of the archaeal 16S rRNA gene and 71% of the archaeal 16S rRNA cDNA sequences (Fig. 3). Results from *mcrA* gene and transcript cDNA sequencing of the control samples also show *Methanosaeta* spp. were the most abundant and active methanogens, representing 86% and 93% of the methanogen community and active methanogen community, respectively. Further, the activity of *Methanosaeta* spp. was reduced in both BES and PA 10 mmol/L inhibition conditions, shown by both 16S rRNA cDNA and *mcrA* transcript cDNA results (Fig. 3B and D). Little difference was observed between *Methanosaeta* spp. activity in PA 0.1 mmol/L compared to the control condition. This is consistent with the methane generation results since, among the four inhibited conditions, the most methane was generated in the PA 0.1 mmol/L treatment (Fig. 2). Results from both the 16S rRNA gene and 16S rRNA cDNA sequencing indicated that *Methanosphaera* spp. and *Methanobrevibacter* spp. represented a greater fraction of the archaeal community and active archaeal community under all inhibited conditions compared to the control (Fig. 3A and B). These genera made up a smaller fraction of the *mcrA*‐based communities, though *Methanobrevibacter* spp. was found to be more active for the most inhibited conditions as compared to the control based on *mcrA* transcript cDNA (Fig. 3C). *Methanoregula* spp. constituted 15–33% of the archaeal community according to 16S rRNA gene sequencing, but its activity represented a much smaller fraction, between 2 and 6%, based on 16S rRNA cDNA sequencing for all conditions. Using *mcrA*‐based sequencing, *Methanoregula* spp. represented less than 2% of the abundance and activity of methanogens under all conditions. Differences between *Methanoregula* 16S rRNA genes and cDNA sequencing have been previously reported (Smith et al. 2015a,b), but little is known about how these levels translate to activity. These results could indicate that *Methanoregula* was present in the inoculum, but not active in the mesocosms or could result from differences in the relationship of activity to rRNA levels within the cells of this genus. Interestingly, *Methanoregula* has only one copy of the 16S rRNA gene, while most other methanogens have two or more. This is further support of the possible different lifestyle strategy of *Methanoregula* compared to other methanogens.

## 16S rRNA cDNA and *mcrA* transcripts highlight differential methanogen response to inhibitors

The mock community results demonstrated that *Methanobacterium* was less abundant in the *mcrA* gene‐based communities compared to the 16S rRNA gene‐based communities (Fig. 1) and this was similarly observed in the mesocosms (Fig. 3A compared to C). However, the RNA‐based sequencing of the *mcrA* transcript cDNA revealed much higher activity of hydrogenotrophic methanogens *Methanobacterium* spp. and *Methanomicrobium* spp. at high PA and both BES conditions compared to the control (Fig. 3D). The 16S rRNA cDNA‐based activity difference for *Methanobacterium* spp. was less substantial, but showed a similar trend (Fig. 3B).

One explanation for this difference in *mcrA*‐based activity may be the presence of a second gene that encodes for an isoenzyme of methyl‐coenzyme M, the *mrtA* gene. This gene has been found in members of both *Methanobacterium* and *Methanomicrobium* genera (Bonacker et al. 1992; Luton et al. 2002), but to date has not been reported in aceticlastic methanogens. Other genera with identified *mrtA* genes include *Methanothermobacter* spp. (GenBank ID AY289753.1) and *Methanosphaera* spp. (Fricke et al. 2006), though the gene is not well annotated or differentiated from reported *mcrA* gene sequences. A comparison between representative sequences from the different operational taxonomic units (OTUs) from this study that were identified as *Methanobacterium* and *Methanomicrobium* shows that of the seven OTUs, one is highly similar (95.9%) to a *Methanobacterium mrtA* gene (OTU 6, Fig. S2) and was highest in relative activity in the BES and PA 10 mmol/L conditions (Fig. S3). Interestingly, pure culture studies with *Methanobacterium thermoautotrophicum* have found differential expression of the *mcrA* and *mrtA* genes, with the *mrtA* being more highly expressed during the exponential growth phase of methanogens and under conditions of high substrate availability (Bonacker et al. 1992; Pihl et al. 1994; Pennings et al. 1997).

The other OTUs observed here were more closely related to known *mcrA* sequences. OTU 2 was also highest in relative activity during methanogenesis inhibited conditions and is more closely related to the *mcrA* gene from *Methanobacterium* sp. T01, which is only 71.8% similar to the *Methanobacterium mrtA* gene. We suspect that there are reasons beyond the increase in *mrtA* expression that allow *Methanobacterium* and *Methanomicrobium* to continue expressing the *mcrA* gene during inhibitor exposure. These findings are consistent with other studies that found hydrogenotrophic methanogens to be less sensitive to inhibition than aceticlastic methanogens (Zinder et al. 1984; Perkins et al. 1994; Xu et al. 2010a; Lins et al. 2015). Multiple explanations have been offered to explain these results, including differences in cell envelopes that might result in differential exposure to inhibitors or differences in coenzyme M transport rates (Xu et al. 2010a).

It is important to note that the shifts in Fig. 3 represent relative changes in total methanogen abundance and activity. Given the challenges with quantitative nucleic acid extractions from heterogeneous biomass samples, these relative abundance and activity data were not converted to an absolute quantification of abundance or activity per biomass. However, by comparing the abundance and activity of methanogens as a fraction of the total community abundance and activity (*Bacteria* and *Archaea*) (Fig. 2), it is clear that the methanogenic activity was lower for higher inhibitor concentrations.

### Activity of syntrophic bacteria *Syntrophomonas* reduced by BES and PA

**Figure 4:**
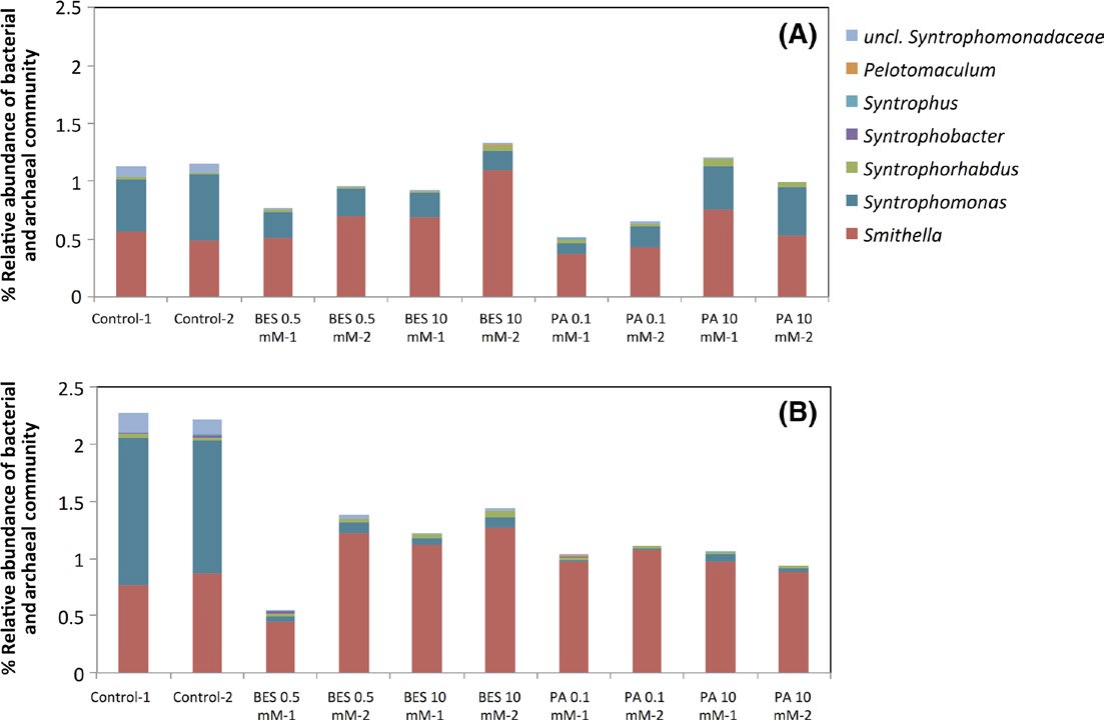
Relative abundance (A) and activity (B) of syntrophic bacteria as a percentage of the total bacterial and archaeal communities based on 16S rRNA gene and 16S rRNA cDNA sequencing in anaerobic mesocosms after 9 days of incubation. Duplicates shown represent duplicate biomass samples from the same reactors.

**Figure 5:**
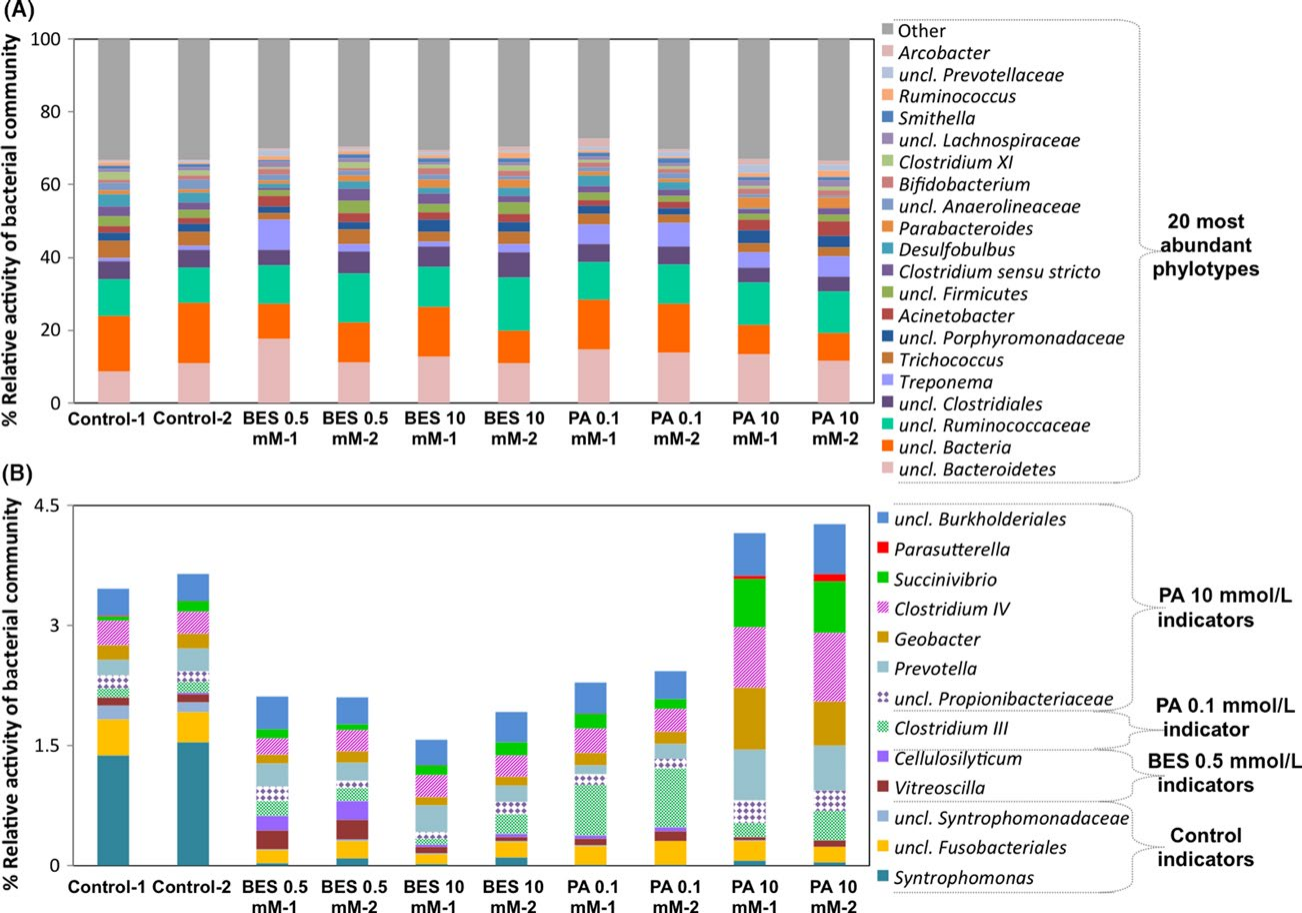
Relative activity based on the 20 most abundant bacterial phylotypes grouped at the genus level (A) and the phylotypes identified as indicator organisms (*P*  < 0.05) (B) in anaerobic mesocosms after 9 days of incubation for each inhibition condition. Duplicates shown represent duplicate biomass samples from the same reactors. No inhibitor was added in control conditions.

Seven populations of previously described syntrophic fatty‐acid oxidizing bacteria were identified in these mesocosm samples. The communities were predominantly comprised of *Syntrophomonas*, a butyrate and higher VFA oxidizer (Sousa et al. 2007), and *Smithella*, a propionate oxidizer (Liu et al. 1999) (Fig. 4). These populations have a coupled metabolism with hydrogenotrophic methanogens to keep the partial pressure of H_2_ low such that their metabolism is energetically favorable. Due to this important relationship between syntrophic bacteria and methanogens, the inhibition of hydrogenotrophic methanogens (Fig. S5) likely caused an increase in the partial pressure of hydrogen and therefore changed the activity of syntrophic bacteria. Differences in gene copy numbers and growth strategies limit the conclusions that can be drawn by using the abundance of 16S rRNA as an indicator of activity (Blazewicz et al. 2013). Therefore, we focus on comparing trends in relative activity within a genus across different treatments, and less on direct comparisons between genera within a specific treatment. Using fluorescence in situ hybridization (FISH) in sewage sludge digesters exposed to BES, Xu et al. (2010b) observed a lower abundance of syntrophic bacteria under methanogenesis‐inhibited conditions compared to a control. In this study, greater changes were observed in relative activity (RNA‐based) as compared to relative abundance (DNA‐based) due to the short duration of the experiment (Fig. 4). The variation in syntrophic bacterial abundance and activity between duplicates was higher in inhibited samples compared to the controls and the differences between other bacterial groups (Fig. 5). This greater variability may be the result of unstable conditions for syntrophic populations as a result of methanogen inhibition. *Syntrophomonas* abundance and activity were lower during inhibited conditions compared to the control (Fig. 4). In contrast, the relative abundance and activity of *Smithella* did not decrease with the presence of either inhibitor. The energetics of butyrate and propionate oxidation is dependent on the partial pressure of hydrogen, which was not measured in this study, but may have contributed to the differential response (Fig. S6). Other factors that may contribute to these observed differences include the production and degradation rates of 16S rRNA levels. While these rates are not known, differences in 16S rRNA gene copy number between *Syntrophomonas* and *Smithella*, three and one copies, respectively, suggest differential growth strategies. Higher 16S rRNA gene copy numbers are associated with higher growth rates following environmental changes (Klappenbach et al. 2000), consistent with our finding that *Syntrophomonas* responded more quickly to the presence of methanogenic inhibitors.

### Inhibitors cause few changes in the 16S rRNA of most active bacterial genera

Overall, the bacterial community present in the mesocosms was quite diverse, containing greater than 9000 OTUs, grouped at a 0.03 sequence similarity cutoff, and 600 phylotypes, grouped based on taxonomic identification at the genus level. The shifts in the structure of the active bacterial community were not significant between duplicates of the different conditions (*θ*_yc_ AMOVA, *P*‐value >0.05) (increasing the number of replicates would have increased the power of this test). There were few changes in the relative activity of the 20 most abundant phylotypes (Fig. 5A). Other studies have found evidence for community shifts during longer term exposure to methanogenic inhibitors, using DGGE and TRFLP following BES exposure for 18 months (Chiu and Lee 2001), 68 days (Lins et al. 2015), and 48 days (Xu et al. 2010b). DGGE also revealed shifts in rumen fluid mesocosms exposed to PA for 24 h when used in combination with other inhibitors (Patra and Yu 2013). It is difficult to compare these previous findings with this study since DGGE and TRFLP provide less resolution for community structure characterization and specific bacterial groups responsible for community shifts were not always identified.

In this study, an indicator analysis (Dufrêne and Legendre 1997) was applied to determine the bacterial populations whose activity (based on 16S rRNA cDNA) was indicative of each condition. The statistically significant groups (*P*‐value <0.05) are shown in Fig. 5B. Of the bacterial populations identified as indicators of the control samples, two are syntrophic populations (*Syntrophomonas* and an unclassified member of *Syntrophomonadaceae*). As previously described, this result was expected due to the inhibition of these groups in both BES and PA conditions. An unclassified member of the order *Fusobacteriales* was also more active in control samples compared to all other conditions. Populations identified as indicators of inhibited conditions include cellulose degraders and bacteria commonly found in rumen and plant matter digesters, including *Cellulosilyticum* (Li et al. 2014), *Clostridium III* and *IV* (Collins et al. 1994), *Prevotella* (Williams et al. 2013), and *Succinivibrio* (Yue et al. 2013). Future studies employing methanogenic inhibitors should recognize the potential for these populations to exhibit increased activity and for the activity of some syntrophic bacteria to decrease.

## Confict of Interest

None declared.

## Supporting information


**Figure S1.** Cumulative gas production of mesocosms.
**Figure S2.** Neighbor‐joining consensus tree of *mcrA* and *mrtA* sequences.
**Figure S3.** Relative abundance and activity of OTUs classified as *Methanobacterium* and *Methanomicrobium*.
**Figure S4.** Relative abundance (DNA) and activity (RNA) of methanogens in duplicate samples from anaerobic mesocosms after 9 days of incubation based on 16S rRNA genes and cDNA (A), and *mcrA* genes and transcript cDNA (B) sequencing.
**Figure S5.** Relative activity of hydrogenotrophic and aceticlastic methanogens based on 16S rRNA cDNA sequencing.
**Figure S6.** Gibb's free energy comparison.
**Table S1.** Primer coverage of the mlas and *mcrA*‐rev primers compared to the modified mlas and *mcrA*‐rev primers used in this study for 32 methanogens for which genomes were available.
**Table S2.** Strains of methanogenic archaea used to create mock communities.
**Table S3.** Comparison of primer match with sequences from methanogens included in mock community.
