# Supplementary material for: Anaerobic microbial community response to methanogenic inhibitors 2‐bromoethanesulfonate and propynoic acid
Source: Microbiologyopen. 2016 Mar 14;5(4):537–50. doi: 10.1002/mbo3.349 (PMC4985588; doi:10.1002/mbo3.349)
Supplement: Supplementary file 1 — Figure S1. Cumulative gas production of mesocosms. Figure S2. Neighbor‐joining consensus tree of mcrA and mrtA sequences. Figure S3. Relative abundance and activity of OTUs classified as Methanobacterium and Methanomicrobium. Figure S4. Relative abundance (DNA) and activity (RNA) of methanogens in duplicate samples from anaerobic mesocosms after 9 days of incubation based on 16S rRNA genes and cDNA (A), and mcrA genes and transcript cDNA (B) sequencing. Figure S5. Relative activity of hydrogenotrophic and aceticlastic methanogens based on 16S rRNA cDNA sequencing. Figure S6. Gibb's free energy comparison. Table S1. Primer coverage of the mlas and mcrA‐rev primers compared to the modified mlas and mcrA‐rev primers used in this study for 32 methanogens for which genomes were available. Table S2. Strains of methanogenic archaea used to create mock communities. Table S3. Comparison of primer match with sequences from methanogens included in mock community. [file MBO3-5-537-s001.docx]

## Supplementary Information

**Anaerobic microbial community response to methanogenic inhibitors 2-bromoethanoesulfonate and propynoic acid**

Tara M. Webster^1^, Adam L. Smith^1^, Raghav R. Reddy^1^, Ameet J. Pinto^2^, Kim F. Hayes^1^, Lutgarde Raskin^1*^

^1^Civil & Environmental Engineering Department, University of Michigan, Ann Arbor, Michigan, USA

^2^Infrastructure and Environment Research Division, School of Engineering, University of Glasgow, Glasgow, United Kingdom

**List of Tables**

Table S1. Primer coverage comparison…………………………………………………………...2

Table S2. Strains of methanogenic archaea used to create mock communities…………………...3

Table S3. Comparison of primer match with sequences from methanogens included in mock community………………………………………………………………………………………...4

**List of Figures**

Figure S1. Cumulative gas production of mesocosms…………………………………………….5

Figure S2. Neighbor-joining consensus tree of *mcrA* and *mrtA* sequences……………………….6

Figure S3. Relative abundance and activity of OTUs classified as *Methanobacterium* and *Methanomicrobium* …………………………………………………………………………….…7

Figure S4. Relative abundance (DNA) and activity (RNA) of methanogens in duplicate samples from anaerobic mesocosms after nine days of incubation based on 16S rRNA genes and cDNA (a) and *mcrA* genes and transcript cDNA (b) sequencing…………………………………………8

Figure S5. Relative activity of hydrogenotrophic and aceticlastic methanogens based on 16S rRNA cDNA sequencing………………………………………………………………………….9

Figure S6. Gibb's free energy comparison……………………………………………………….10

Table S1. Primer coverage of the mlas and mcrA-rev primers compared to the modified mlas and mcrA-rev primers used in this study for 32 methanogens for which genomes were available. Primer pair coverage is a score based on the number of base pairs of the forward and reverse primers that match the template sequence, normalized to the length of the primers. These scores were generated with MFE Primer 2.0 ([Qu et al., 2012](#_ENREF_51)). The maximum 100% coverage would result from primers that are the same length and without mismatches to the template. A primer pair coverage cutoff value of 30 was used.

| NCBI Reference | Taxonomy | Size (bp) | Primer Pair Coverage (%) | |
| --- | --- | --- | --- | --- |
|  |  |  | mlas/ | modified mlas/ mcrA-rev |
|  |  |  | mcrA-rev |  |
| NC_015574.1 | *Methanobacterium* sp. SWAN-1 | 472 | 97 | 97 |
| NC_009135.1 | *Methanococcus maripaludis* | 469 | 97 | 97 |
| NC_009634.1 | *Methanococcus vannielii* | 469 | 97 | 97 |
| NC_009637.1 | *Methanococcus maripaludis* | 469 | 97 | 97 |
| NC_009975.1 | *Methanococcus maripaludis* | 469 | 97 | 97 |
| NC_003552.1 | *Methanosarcina acetivorans* | 490 | 97 | 97 |
| NC_003901.1 | *Methanosarcina mazei* | 490 | 97 | 97 |
| NC_014408.1 | *Methanothermobacter marburgensis* | 469 | 97 | 97 |
| NC_014507.1 | *Methanoplanus petrolearius* | 493 | 97 | 97 |
| NC_007355.1 | *Methanosarcina barkeri* | 490 | 97 | 97 |
| NC_007681.1 | *Methanosphaera stadtmanae* | 469 | 97 | 97 |
| **NC_009051.1** | ***Methanoculleus marisnigri*** | **493** | **75.8** | **97** |
| **NC_009712.1** | ***Methanoregula boonei*** | **472** | **75.8** | **97** |
| **NC_015416.1** | ***Methanosaeta concilii*** | **472** | **75.8** | **97** |
| **NC_009515.1** | ***Methanobrevibacter smithii*** | **472** | **<30** | **97** |
| **NC_009464.1** | ***Methanocella arvoryzae*** | **469** | **<30** | **97** |
| **NC_015847.1** | ***Methanococcus maripaludis*** | **469** | **<30** | **97** |
| **NC_017527.1** | ***Methanosaeta harundinaceae*** | **472** | **<30** | **97** |
| NC_018227.1 | *Methanoculleus bourgensis* | 493 | 97 | 97 |
| NC_018876.1 | *Methanolobus psychrophilus* | 490 | 97 | 97 |
| **NC_019943.1** | ***Methanoregula formicicum*** | **472** | **33** | **97** |
| **NC_005791.1** | ***Methanococcus maripaludis*** | **469** | **<30** | **97** |
| NC_023044.1 | *Methanobacterium* sp. MB1 | 469 | 89 | 89 |
| NC_007955.1 | *Methanococcoides burtonii* | 490 | 56 | 56 |
| NC_008553.1 | *Methanosaeta thermophila* | 472 | 56 | 56 |
| NC_019977.1 | *Methanomethylovorans hollandica* | 490 | 38 | 38 |
| NC_008942.1 | *Methanocorpusculum labreanum* | 493 | 33 | 33 |
| NC_015216.1 | *Methanobacterium* sp. AL-21 | 466 | 33 | 33 |
| NC_011832.1 | *Methanosphaerula palustris* | 472 | 33 | 33 |
| NC_014222.1 | *Methanococcus voltae* | 469 | 33 | 33 |
| NC_021355.1 | *Methanobrevibacter* sp. AbM4 | 472 | 33 | 33 |
| **NC_020389.1** | ***Methanosarcina mazei*** | **490** | **<30** | **33** |

Table S2. Strains of methanogenic archaea used to create mock communities.

| Strain ID | Organism name | 16S rRNA gene copy number | *mcrA* gene copy number |
| --- | --- | --- | --- |
| **DSM-862** | *Methanobacterium bryantii*^ǂ^ | 2.5 | 2 |
| **DSM-861** | *Methanobrevibacter smithii* | 2 | 1 |
| **DSM-2067** | *Methanococcus maripaludis* | 3 | 1 |
| **DSM-3671** | *Methanosaeta concilii* | 2 | 1 |
| **DSM-2834** | *Methanosarcina acetivorans* | 3 | 1 |
| **DSM-800** | *Methanosarcina barkeri* | 3 | 1 |
| **DSM-3091** | *Methanosphaera stadtmanae* | 4 | 1 |
| **DSM-1101** | *Methanospirillum hungatei* | 4 | 1 |
| **DSM-864** | *Methanospirillum hungatei* | 4 | 1 |
| **DSM-1053** | *Methanothermobacter thermautotrophicus* | 2 | 2 |

^ǂ^ Gene copy numbers for *Methanobacterium bryantii* were estimated based on the complete ge

Table S3. Comparison of primer match with sequences from methanogens included in mock community

| Organism | mcrA-forward primer (modified mlas primer (Steinberg and Regan, 2009))  **5’-GGYGGTGTMGGNTTCACHCARTA-3’** |
| --- | --- |
| ***Methanospirillum hungatei*** | GGTGGTGTCGGATT**T**ACCCAGTA |
| ***Methanobacterium sp. AL*** | GGTGGTGTAGGTTTCACACAGTA |
| ***Methanobacterium sp. AL*** | GG**A**GG**A**GTAGGATTCACTCAGTA |
| ***Methanobacterium sp. SWAN 1*** | GGCGGTGTCGGTTTCACCCAGTA |
| ***Methanobacterium sp. SWAN 1*** | GGTGGTGT**T**GGATTCACACAGTA |
| ***Methanobrevibacter smithii*** | GGTGGTGTAGGATTCACTCAATA |
| ***Methanothermobacter thermautotrophicus*** | GGTGGTGTAGGATTCACCCAGTA |
| ***Methanothermobacter thermautotrophicus*** | GGTGGTGT**G**GGTTTCACCCAGTA |
| ***Methanosphaera stadtmanae*** | GGTGGTGTAGGATTCACACAATA |
| ***Methanococcus maripaludis*** | GGTGGTGTAGGATTCACACAATA |
| ***Methanosaeta concilii*** | GGTGGTGTAGGTTTCACACAGTA |
| ***Methanosarcina acetivorans*** | GGTGGTGTCGGGTTCACCCAGTA |
| ***Methanosarcina barkeri*** | GGTGGTGTCGGATTCACACAGTA |

| Organism | mcrA-reverse primer  (Steinberg and Regan, 2008)  **5’-CGTTCATBGCGTAGTTVGGRTAGT-3’** |
| --- | --- |
| ***Methanospirillum hungatei*** | CGTTCATTGCGTAGTTCGGGTAGT |
| ***Methanobacterium sp. AL*** | CGTTCATTGC**A**TAGTTAGGGTAGT |
| ***Methanobacterium sp. AL*** | CGTTCATTGCGTAGTT**T**GGATAGT |
| ***Methanobacterium sp. SWAN 1*** | CGTTCATGGCGTAGTTCGGGTAGT |
| ***Methanobacterium sp. SWAN 1*** | CGTTCATTGCGTAGTTAGGGTAGT |
| ***Methanobrevibacter smithii*** | CGTTCATTGCGTAGTTAGGGTAGT |
| ***Methanothermobacter thermautotrophicus*** | CGTTCATGGCGTAGTT**T**GGATAGT |
| ***Methanothermobacter thermautotrophicus*** | CGTTCATGGCGTAGTT**T**GGATAGT |
| ***Methanosphaera stadtmanae*** | CGTTCATTGCGTAGTTAGGGTAGT |
| ***Methanococcus maripaludis*** | CGTTCATTGCGTAGTTAGGGTAGT |
| ***Methanosaeta concilii*** | CGTTCATGGCGTAGTTCGGGTAGT |
| ***Methanosarcina acetivorans*** | CGTTCATTGCGTAGTTCGGGTAGT |
| ***Methanosarcina barkeri*** | CGTTCATTGCGTAGTTGGGGTAGT |

Primer sequences were used to search for matching sequences in the complete genomes of strains included in the mock communities. Complete genomes were downloaded from the Joint Genome Institute and NCBI. Mismatches to primers are shown in bolded red font. There were no mismatches to the 16S rRNA gene forward and reverse primers used. Two copies of the *mcrA* gene are present in *Methanobacterium* and *Methanothermobacter*.


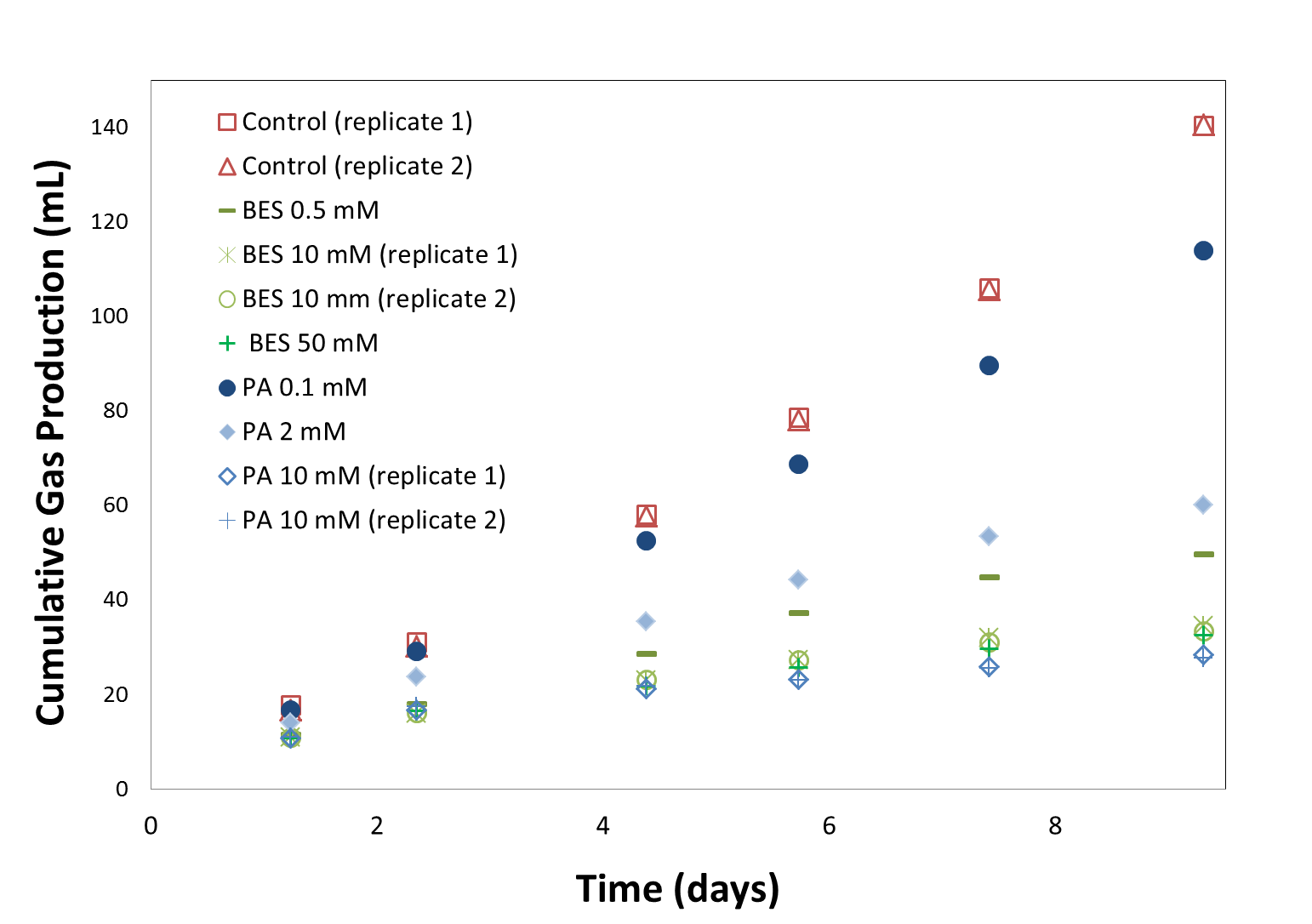


Figure S1. Cumulative gas production for all inhibitor concentrations tested including replicate mesocosms.


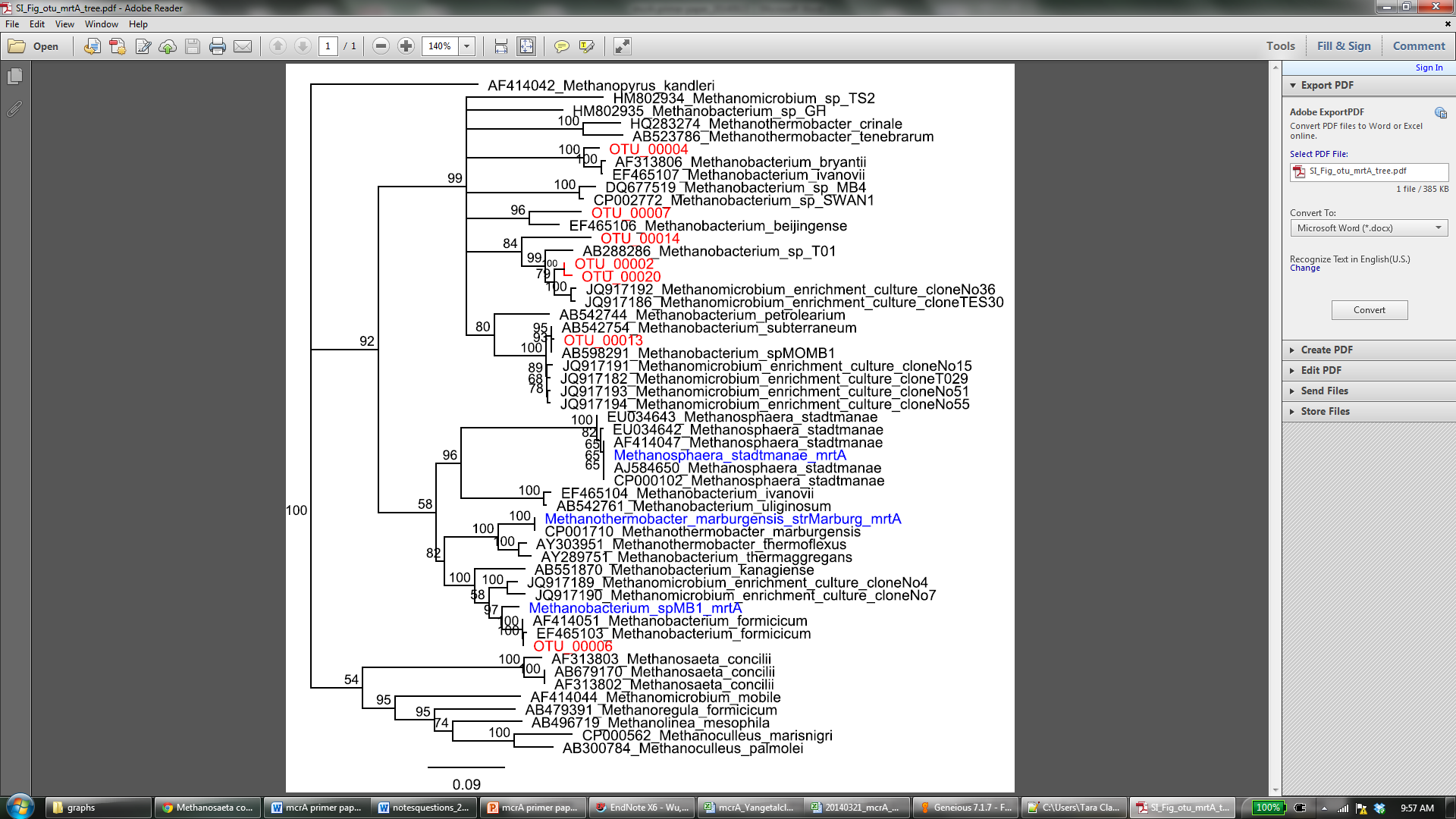


Figure S2. Neighbor-joining consensus tree created using Geneious version 7 (Kearse et al., 2012) based on *mcrA* sequences aligned with MUSCLE, showing sequences used for taxonomic identification (black), *mrtA* sequences (blue), and representative sequences from OTUs identified as *Methanobacterium* and *Methanomicrobium* (red). *Methanopyrus kandleri* was selected as the out group. The *mrtA* sequences included the only *mrtA* sequences annotated in NCBI.


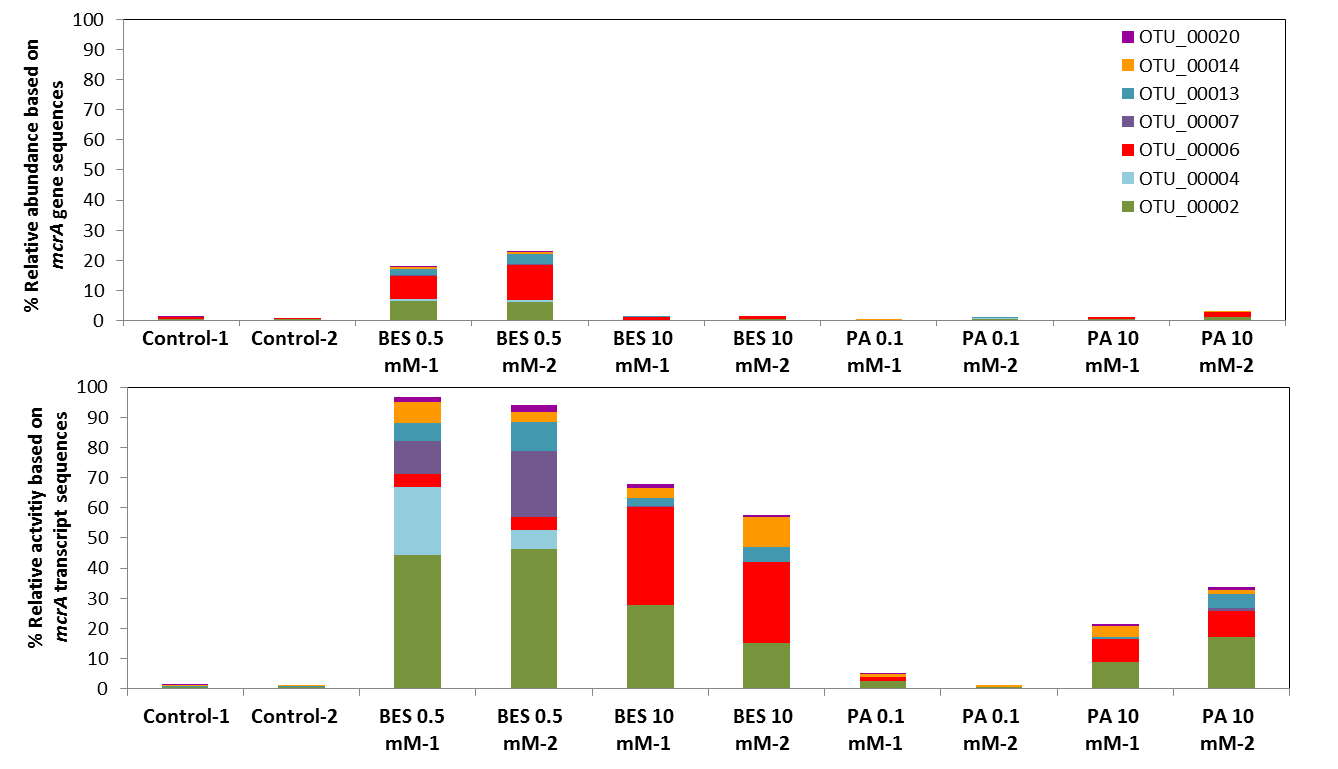


Figure S3. Relative abundance (top) and activity (bottom) of OTUs classified as *Methanobacterium* and *Methanomicrobium* based on *mcrA* gene and *mcrA* transcript cDNA sequences. Results from duplicate biomass samples from each mesocosm are shown.

Figure S4. Relative abundance (DNA) and activity (RNA) of methanogens in duplicate samples from anaerobic mesocosms after nine days of incubation based on 16S rRNA genes and cDNA (a) and *mcrA* genes and transcript cDNA (b) sequencing.


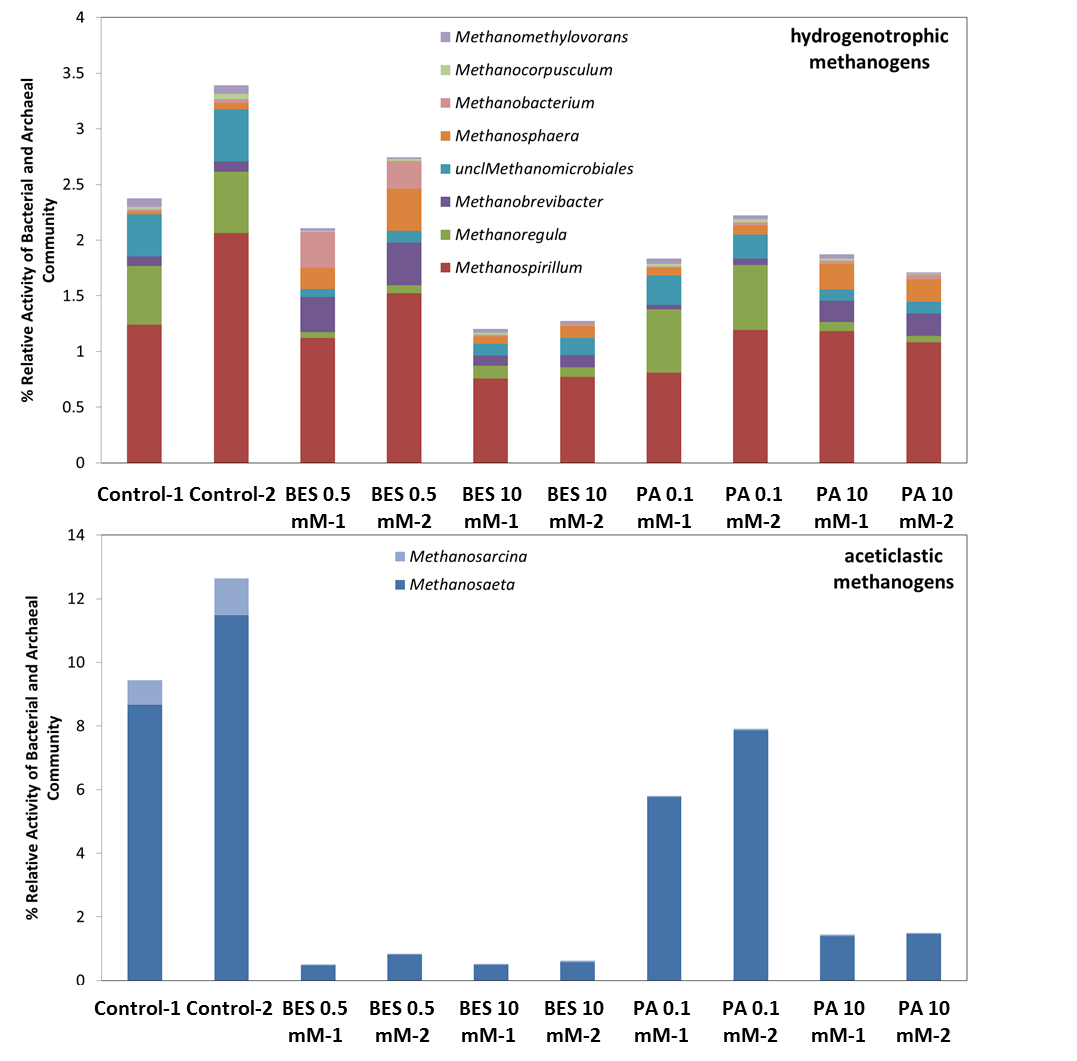


Figure S5. Relative activity of hydrogenotrophic (top) and aceticlastic (bottom) methanogens based on 16S rRNA cDNA sequencing.

Figure S6. Gibb’s free energy (∆G) versus the partial pressure of hydrogen (P_H2_) for “propionate oxidation via butyrate”, in which propionate is dismutated by *Smithella* spp. to acetate and butyrate followed by butyrate oxidation by *Syntrophomonas* spp. (Gan *et al.,* 2012), “classical propionate oxidation”, in which propionate oxidation occurs by *Smithella* spp., and “butyrate oxidation”, in which butyrate oxidation occurs directly by *Syntrophomonas* spp. The ∆G_reaction_ were calculated based on the free energies of formation (∆G_f_°) in (Madigan et al., 2010) and assumed to be 48,400 J/mol, 76,500 J/mol, and 48,300 J/mol for the Smithella, Classical, and Syntrophomonas Pathways, respectively, shown below. Temperature was assumed to be 31°C. Concentrations of acetate, propionate, butyrate, and bicarbonate were assumed to be 22.9, 12.2, 6.43, and 49.2 mM, respectively. A pH of 6.5 was assumed. A ∆G<0 indicates an exergonic reaction.

Propionate Oxidation via Butyrate

$$2CH_{3}CH_{2}COO^{-}+2H_{2}O \to3CH_{3}COO^{-}+2H_{2}+H^{+}$$

Classical Propionate Oxidation

$$CH_{3}CH_{2}COO^{-}+3H_{2}O \to CH_{3}COO^{-}+HCO_{3}^{-}+3H_{2}+H^{+}$$

Butyrate Oxidation

$$CH_{3}CH_{2}CH_{2}COO^{-}+2H_{2}O \to2CH_{3}COO^{-}+2H_{2}+H^{+}$$

**References**

Gan, Y., Q. Qiu, P. Liu, J. Rui and Y. Lu (2012). Syntrophic Oxidation of Propionate in Rice Field Soil at 15 and 30°C under Methanogenic Conditions. *Appl. Environ. Microbiol.* 78(14): 4923-4932.

Kearse, M., R. Moir, A. Wilson, S. Stones-Havas, M. Cheung, S. Sturrock, S. Buxton, A. Cooper, S. Markowitz and C. Duran (2012). Geneious Basic: an integrated and extendable desktop software platform for the organization and analysis of sequence data. *Bioinformatics* 28(12): 1647-1649.

Madigan, M. T., J. M. Martinko, P. V. Dunlap and D. P. Clark (2010). *Brock Biology of Microorganisms*. 13th ed. Boston, Benjamin Cummings.

Smith, A. L., S. J. Skerlos and L. Raskin (2013). Psychrophilic anaerobic membrane bioreactor treatment of domestic wastewater. *Water Res*. 47: 1655-1665.

Steinberg, L. M. and J. M. Regan (2008). Phylogenetic Comparison of the Methanogenic Communities from an Acidic, Oligotrophic Fen and an Anaerobic Digester Treating Municipal Wastewater Sludge. *Appl. Environ. Microbiol*. 74(21): 6663-6671.

Steinberg, L. M. and J. M. Regan (2009). mcrA-Targeted Real-Time Quantitative PCR Method To Examine Methanogen Communities. *Appl. Environ. Microbiol*. 75(13): 4435-4442.
